# Association of Glycated Hemoglobin Levels With Risk of Pancreatic Cancer

**DOI:** 10.1001/jamanetworkopen.2020.4945

**Published:** 2020-06-12

**Authors:** Bechien U. Wu, Rebecca K. Butler, Eva Lustigova, Jean M. Lawrence, Wansu Chen

**Affiliations:** 1 Center for Pancreatic Care, Division of Gastroenterology, Kaiser Permanente Los Angeles Medical Center, Los Angeles, California; 2 Department of Research and Evaluation, Kaiser Permanente Southern California, Pasadena

## Abstract

**Importance:**

New-onset diabetes after the age of 50 years is a potential indicator of pancreatic cancer. Understanding the associations between hyperglycemia, diabetes, and pancreatic cancer, including pancreatic ductal adenocarcinoma, is key to developing an approach to early detection.

**Objective:**

To assess the association of elevation in glycated hemoglobin (HbA_1c_) with the risk of pancreatic cancer.

**Design, Setting, and Participants:**

This cohort study was conducted using data collected from an integrated health care system in California. A total of 851 402 patients aged 50 to 84 years who had HbA_1c_ measurements taken between 2010 and 2014 were identified as the base cohort, with 12 contemporaneous cohorts created based on varying HbA_1c_ thresholds (ie, 6.1%, 6.3%, 6.5%, and 6.7%) and prior diabetes status. Data analysis was conducted from August 2018 to September 2019.

**Main Outcomes and Measures:**

New cases of pancreatic cancer identified through cancer registry and California death files during a 3-year period. Three-year risk, incidence rate, sensitivity, number of patients needed to screen to detect 1 case, timing, and stage at diagnosis were determined.

**Results:**

Among 851 402 patients in the base cohort, 447 502 (52.5%) were women, 255 441 (30.0%) were Hispanic participants, 383 685 (45.1%) were non-Hispanic white participants, 100 477 (11.8%) were Asian participants, and 88 969 (10.4%) were non-Hispanic black participants, with a median (interquartile range) age of 62 (56-69) years and a median (interquartile range) HbA_1c_ level of 6.0% (5.7%-6.6%). The incidence rate of pancreatic cancer was 0.45 (95% CI, 0.43-0.49) per 1000 person-years. After excluding prior diabetes as well as confirmation of new-onset hyperglycemia based on an HbA_1c_ level of 6.5%, a total of 20 012 patients remained, with 74 of 1041 pancreatic ductal adenocarcinoma cases (7.1%) from the base cohort included. The rate of pancreatic cancer was 0.72 (95% CI, 0.32-1.42) per 1000 person-years among Asian patients, 0.83 (95% CI, 0.35-1.71) per 1000 person-years among non-Hispanic black patients, 0.84 (95% CI, 0.48-1.37) per 1000 person-years among Hispanic patients, and 2.37 (95% CI, 1.75-3.14) per 1000 person-years among non-Hispanic white patients. Overall, 42 of 74 cancers (56.8%) were diagnosed within 1 year of the index laboratory test. Among 1041 total cases, 708 (68.0%) had staging information available, of whom 465 (65.7%) had stage III or IV disease at diagnosis. In the base cohort, the number needed to undergo evaluation to identify a single case of pancreatic ductal adenocarcinoma was 818 (95% CI, 770-869), with estimates ranging from 206 (95% CI, 160-264) to 600 (95% CI, 540-666) in the subcohorts.

**Conclusions and Relevance:**

The findings of this study suggest that screening patients for pancreatic cancer based solely on elevation in HbA_1c_ level is unlikely to represent an effective strategy. Future efforts to identify a high-risk population based on changes in glycemic parameters should account for racial/ethnic differences.

## Key Points

**Question:**

What is the association of elevated glycated hemoglobin levels with the risk of pancreatic cancer?

**Findings:**

In this cohort study with 851 402 participants, risk of pancreatic cancer varied in association with prior diabetes status as well as glycated hemoglobin level; however, this risk was not consistently observed across racial/ethnic groups. The number of patients who need to undergo further investigation to identify a single pancreatic cancer ranged from 206 to 600.

**Meaning:**

In this study, the risk of pancreatic cancer associated with a newly identified elevation in blood glucose varied by race/ethnicity and did not reach a level sufficient to justify potential widespread screening.

## Teaser

This cohort study assesses the association of elevation in glycated hemoglobin level with the risk of pancreatic cancer.

## Introduction

Pancreatic cancer is the third leading cause of cancer-related death in the United States, with a 5-year survival rate below 10%.^1^ A contributor to poor survival for individuals who develop pancreatic cancer is the advanced stage of disease at the time of clinical presentation. The ability to detect pancreatic cancer at an earlier stage represents an important opportunity to potentially improve outcomes. However, because of the relatively low prevalence of pancreatic cancer (12.9 per 100 000 person-years [PYs]),^2^ the United States Preventive Services Task Force does not recommend population-based screening.^3^ As a result, targeted screening for patients from high-risk population subgroups has emerged as the most promising approach for early detection.

Hyperglycemia has been identified in patients with pancreatic cancer up to 36 months before cancer diagnosis.^4^ Newly diagnosed (ie, incident) diabetes after the age of 50 years has received increasing attention as a potential marker of undiagnosed pancreatic cancer,^5^ with 3-year cancer rates ranging from 0.25% to 1.0%^6,7,8^ and the highest incidence reported in studies that have relied on glycemic criteria^7,8^ rather than physician diagnosis.^6^ However, it remains unclear to what extent glycemic parameters, particularly glycated hemoglobin (HbA_1c_) levels, are associated with overall risk of pancreatic cancer.

The objective of the present study was to evaluate the association of glycemic abnormality and risk of pancreatic cancer. We hypothesized that applying criteria other than those traditionally used in the definition of diabetes could lead to improved sensitivity for early detection of patients with pancreatic cancer. Therefore, we sought to characterize the performance of various thresholds of HbA_1c_ level, the most common test used to diagnosis diabetes, in a series of comparative cohort studies.

## Methods

The present study was approved by the institutional review board of Kaiser Permanente Southern California (KPSC) with a waiver of informed consent because the study did not involve direct patient contact. This study is reported in accordance with the Strengthening the Reporting of Observational Studies in Epidemiology (STROBE) reporting guideline.^9^

### Study Design and Setting

We performed a series of retrospective cohort studies to assess the association of varying glycemic criteria with the risk of pancreatic cancer. Analysis was performed on data collected from the research data warehouse of KPSC. Cohort entry was between January 1, 2010, and December 31, 2014, with follow-up through December 31, 2017. Kaiser Permanente Southern California is a community-based integrated health care system providing comprehensive care to 4.6 million enrollees. The full spectrum of health care services provided to health plan enrollees includes ambulatory care as well as acute hospital care, imaging services, and pharmacy services. The beginning of the study period was selected based on a shift in practice in favor of HbA_1c_ levels following revised guidelines from the American Diabetic Association that incorporated HbA_1c_ levels as part of the diagnostic criteria for diabetes.^10^

### Identification of Study Cohorts

We first identified patients aged 50 to 84 years who had at least 1 measure of HbA_1c_ between 2010 and 2014 in a KPSC medical facility (referred to as the base cohort). This age range was selected to identify a study cohort that would be representative of a potentially suitable population to undergo screening for early detection of pancreatic cancer. Blood samples collected in outpatient, emergency department, and inpatient settings were included. Point-of-care testing for HbA_1c_ levels was rarely performed during the study period and not included in the analysis. For each patient, the first laboratory test that met the entry criteria was referred to as the index HbA_1c_ test, and the date of the test was referred to as the index date. For the purpose of comparison, the following 12 contemporaneous cohorts were created. Patients with an HbA_1c_ level at or above prespecified thresholds (ie, 6.1%, 6.3%, 6.5% and 6.7% of total hemoglobin [to convert to proportion of total hemoglobin, multiply by 0.01]) during the study period, irrespective of previous values or diabetes status, were included in 4 separate elevated HbA_1c_ (EGH) cohorts, referred to as EGH_6.1%_, EGH_6.3%_, EGH_6.5%_, and EGH_6.7%_. These thresholds for HbA_1c_ level were selected a priori to provide a range of values across the spectrum of patients likely to be experiencing prediabetes or recent-onset diabetes. We created 4 diabetes-excluded cohorts (DECs), which included patients from each EGH cohort with no history of diabetes (extending back to 2000, when data were first available) before the index date based on a previously validated algorithm for identification of patients with diabetes. This algorithm defined diabetes based on diagnosis codes, use of diabetes medication, or elevated glycemic laboratory values. We used the following logic to identify and exclude patients with history of diabetes: any hospital discharge code for diabetes (*International Classification of Diseases, Ninth Revision* [*ICD*-*9*] code 250.XX), any KPSC internal code for diabetes (ie, 200, 1201, 1202, 1203, 1204, 1839, 3141, 3186, 3639, 4124, or 5782), any prior HbA_1c_ level greater than 7.0%, or any dispensing record for insulin or an oral hypoglycemic medication (not including metformin). These cohorts were designated DEC_6.1%_, DEC_6.3%_, DEC_6.5%_, and DEC_6.7%_. Finally, we created confirmed index hyperglycemia cohorts (IHCs). To evaluate a potential increase in positive predictive value by adopting more stringent criteria defining patients with new-onset hyperglycemia, we established an additional series of patient cohorts including only patients with a confirmed finding of newly elevated HbA_1c_. Confirmation of newly elevated HbA_1c_level was performed by requiring a test below the respective threshold in the 18 months before the index date. The criteria defining IHCs were selected to reflect those of an ongoing prospective cohort study.^11^ These cohorts were designated IHC_6.1%_, IHC_6.3%_, IHC_6.5%_, and IHC_6.7%_. For all study cohorts, patients with history of pancreatic cancer before the index date either based on diagnosis code (*ICD-9* code 157.x or *ICD*-*10* code C25.x) or KPSC Cancer Registry^12^ and patients not enrolled in the health plan on the index date were excluded.

### Outcome Definition

The primary outcome, pancreatic cancer, was ascertained through the KPSC Cancer Registry^12^ and linkage of enrollees with decedents from the California death files.^13^ The cases identified through the Cancer Registry were pancreatic ductal adenocarcinoma (PDAC) based on specific histology codes. However, such a restriction was not applied to the cases from the California death files because the information on histology was not available.

### Statistical Analysis

For each of the 12 cohorts, the following analyses were performed. We first estimated the 3-year risk and rate of pancreatic cancer per 1000 PYs and their 95% CIs. Then we calculated the number of patients needed to screen to detect 1 PDAC case. Among patients diagnosed with or who died of pancreatic cancer, we examined time to cancer with 2 different approaches, as follows: median (interquartile range [IQR]) follow-up time and cumulative percentage diagnosed or died in the first, second, and third years. Finally, we reported the distribution of cancer stage at the time of diagnosis. Analyses were conducted in SAS statistical software version 9.4 (SAS Institute). All analyses were descriptive in nature. We did not perform hypothesis testing and thus did not prespecify a level of statistical significance.

## Results

### Eligible Study Participants 

Among the base cohort of 851 402 patients, 447 502 (52.5%) were women, 255 441 (30.0%) were Hispanic participants, 383 685 (45.1%) were non-Hispanic white participants, 100 477 (11.8%) were Asian participants, and 88 969 (10.4%) were non-Hispanic black participants, with a median (IQR) age of 62 (56-69) years and a median (IQR) HbA_1c_ level of 6.0% (5.7%-6.6%) (Table 1). The number of unique eligible study participants included in the EGH cohort, DEC, and IHC with varying HbA_1c_ thresholds can be found in the Figure. After excluding prior diabetes as well as confirmation of new-onset hyperglycemia based on an HbA_1c_ level of 6.5%, a total of 20 012 patients remained in the IHC_6.5%_ cohort, with 74 of 1041 PDAC cases (7.1%) from the base cohort included. The cohort size ranged from a high of 495 310 patients (58.2% of the base cohort) for EGH_6.1%_ to a low of 12 540 (1.5% of the base cohort) for IHC_6.7%._

**Table 1.** Patient Characteristics by Cohort Type

Characteristic	No. (%)																		
	Base cohort (any)	Elevated HbA_1c_ cohort						Diabetes-excluded cohort							Confirmed index hyperglycemia cohort				
		6.1%		6.3%		6.5%	6.7%	6.1%	6.3%		6.5%		6.7%		6.1%	6.3%		6.5%	6.7%
No.	851 402	495 310	395 304		331 609		291 842	210 539		134 815		89 704		62 741	34 881		27 825	20 012	12 540
Age at index, median (IQR), y	62 (56-69)	62 (56-70)	62 (56-69)		62 (56-69)		62 (55-69)	62 (56-68)		62 (56-68)		61 (56-67)		61 (55-67)	64 (57-71)		64 (57-71)	64 (57-71)	63 (57-70)
Women	447 052 (52.5)	253 007 (51.1)	197 011 (49.8)		161 343 (48.7)		139 331 (47.7)	114 980 (54.6)		72 009 (53.4)		46 251 (51.6)		30 895 (49.2)	19 103 (54.8)		15 344 (55.1)	10 932 (54.6)	6534 (52.1)
Race/ethnicity																			
Asian, non-Hispanic	100 477 (11.8)	70 580 (14.2)	56 544 (14.3)		46 593 (14.1)		40 065 (13.7)	32 195 (15.3)		21 658 (16.1)		14 470 (16.1)		9645 (15.4)	5381 (15.4)		4897 (17.6)	3749 (18.7)	2276 (18.1)
Black, non-Hispanic	88 969 (10.4)	61 472 (12.4)	49 960 (12.6)		41 374 (12.5)		35 743 (12.2)	25 025 (11.9)		16 965 (12.6)		11 067 (12.3)		7337 (11.7)	3491 (10.0)		3373 (12.1)	2636 (13.2)	1683 (13.4)
Hispanic	255 441 (30.0)	174 218 (35.2)	142 665 (36.1)		122 183 (36.8)		109 530 (37.5)	72 128 (34.3)		47 457 (35.2)		32 294 (36.0)		23 403 (37.3)	10 588 (30.4)		8679 (31.2)	6181 (30.9)	3880 (30.9)
White, non-Hispanic	383 685 (45.1)	176 284 (35.6)	135 977 (34.4)		112 941 (34.1)		99 041 (33.9)	75 397 (35.8)		44 961 (33.4)		29 257 (32.6)		20 449 (32.6)	14 743 (42.3)		10 336 (37.1)	7073 (35.3)	4476 (35.7)
Other or unknown	22 830 (2.7)	12 756 (2.6)	10 158 (2.6)		8518 (2.6)		7463 (2.6)	5794 (2.8)		3774 (2.8)		2616 (2.9)		1907 (3.0)	678 (1.9)		540 (1.9)	373 (1.9)	225 (1.8)
HbA_1c_, median (IQR), % of total hemoglobin	6.0 (5.7-6.6)	6.6 (6.2-7.6)	6.9 (6.4-8.0)		7.2 (6.7-8.3)		7.4 (6.9-8.6)	6.3 (6.1-6.6)		6.5 (6.3-6.9)		6.8 (6.6-7.5)		7.1 (6.8-8.3)	6.1 (6.1-6.2)		6.3 (6.3-6.5)	6.6 (6.5-6.7)	6.8 (6.7-7.0)
Acute pancreatitis^a^	11 566 (1.4)	8087 (1.6)	6887 (1.7)		6032 (1.8)		5493 (1.9)	2234 (1.1)		1497 (1.1)		952 (1.1)		654 (1.0)	531 (1.5)		459 (1.6)	301 (1.5)	204 (1.6)
Chronic pancreatitis^a^	2407 (0.3)	1783 (0.4)	1578 (0.4)		1407 (0.4)		1286 (0.4)	399 (0.2)		284 (0.2)		188 (0.2)		123 (0.2)	90 (0.3)		107 (0.4)	82 (0.4)	52 (0.4)
Pancreatic cyst^a^	2025 (0.2)	1405 (0.3)	1212 (0.3)		1043 (0.3)		941 (0.3)	446 (0.2)		320 (0.2)		211 (0.2)		139 (0.2)	119 (0.3)		128 (0.5)	87 (0.4)	68 (0.5)
Alcohol use disorder^a^	43 041 (5.1)	22 299 (4.5)	17 853 (4.5)		15 083 (4.5)		13 372 (4.6)	8244 (3.9)		5102 (3.8)		3280 (3.7)		2283 (3.6)	1884 (5.4)		1401 (5.0)	956 (4.8)	632 (5.0)

Abbreviation: HbA_1c_, glycated hemoglobin; IQR, interquartile range.

SI conversion factor: To convert HbA_1c_ to proportion of total hemoglobin, multiply by 0.01.

^a^ Acute pancreatitis, chronic pancreatitis, pancreatic cyst, and alcohol use disorder: any diagnosis prior to index date.

**Figure.**
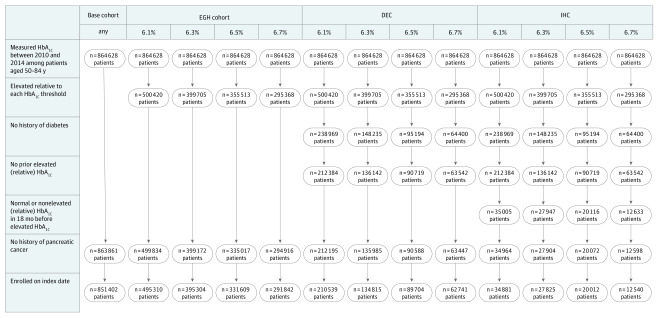
Cohort Assembly DEC indicates diabetes-excluded cohort; EGH, elevated glycated hemoglobin; HbA_1c_, glycated hemoglobin; and IHC, index hyperglycemia cohort.

### Patient Characteristics

Patients in the EGH cohort and DEC had similar median ages (61-62 years) compared with the base cohort (62 years of age) (Table 1). However, patients in the IHC tended to be 1 to 2 years older (median age, 63-64 years) compared with patients in the base cohort, EGH cohort, and DEC (Table 1). In addition, the IHC appeared to have similar or higher frequency of women (52.1%-55.1%) compared with patients in the base cohort (52.5%). There was variation in racial/ethnic composition across the study cohorts, with a lower proportion of white patients in the analytic cohorts compared with the base cohort. Both Hispanic and non-Hispanic Asian patients appeared to be more frequent in the EGH cohort, DEC, and IHC (Hispanic participants in EGH cohort, 35.2%-36.5%; DEC, 34.3%-37.3%; IHC, 30.4%-31.2%; Asian participants in EGH cohort, 13.7%-14.3%; DEC, 15.3%-16.1%; IHC, 15.4%-18.7%) compared with the corresponding frequencies in the base cohort (Hispanic participants in base cohort, 255 441 [30.0%]; Asian participants, 100 477 [11.8%]). In contrast, the proportions of non-Hispanic white patients seemed to be much lower in the EGH cohort, DEC, and IHC compared with the base cohort (EGH, 33.9%-35.6%; DEC, 32.6%-35.8%; IHC, 35.7%-42.3%; base, 383 685 [45.1%]). In addition, for a given threshold for HbA_1c_ level, the observed median HbA_1c_ value at cohort entry varied based on entry criteria, with the highest observed level among the EGH cohort (eg, median [IQR] HbA_1c_ level, 7.2% [6.7%-8.3%]) was for patients in EGH_6.5%_, and the lowest in the ICH (eg, median [IQR] HbA_1c_ level, 6.6% [6.5%-6.7%]) was for patients in ICH_6.5%_ (Table 1).

### Risk and Rate of PDAC

In the base cohort, the 3-year risk and the incidence rate of pancreatic cancer were 0.12% (95% CI, 0.12%-0.13%) and 0.45 (95% CI, 0.43-0.49) per 1000 PYs, respectively. The estimated 3-year risk and incidence rates per 1000 PYs for each cohort are reported in Table 2. The incidence rates of 0.64 (0.59-0.68) in EGH_6.1%_ and 0.83 (0.77-0.90) in EGH_6.7%_ are 1.4- and 1.8-fold higher than the incidence rate of 0.46 (0.43-0.49) of the base cohort. The incidence rates of 0.78 (0.61-0.98) in IGH_6.1%_ and 1.82 (1.39-2.33) in IGH_6.7%_ are 1.7- and 4.0-fold higher than the incidence rate of 0.46 (0.43-0.49) in the base cohort. Both risk and incidence rates increased with more stringent eligibility criteria (ie, from EGH cohort to DEC to IHC) as well as with higher thresholds for HbA_1c_ levels (ie, from 6.1% to 6.3% to 6.5% to 6.7%). The 3-year PDAC risk ranged from 0.21% (0.20%-0.23%) in patients in EGH_6.1%_ to 0.80% (0.65%-0.99%) in patients in IHC_6.7%_. The rate of pancreatic cancer varied by race/ethnicity from 0.72 (95% CI, 0.32-1.42) per 1000 PYs among Asian patients, 0.83 (95% CI, 0.35-1.71) per 1000 PYs among non-Hispanic black patients, 0.84 (95% CI, 0.48-1.37) per 1000 PYs among Hispanic patients, and 2.37 (95% CI, 1.75-3.14) per 1000 PYs among non-Hispanic white patients.

**Table 2.** Rate, Prevalence, and Risk of Pancreatic Ductal Adenocarcinoma, by Cohort

Cohort	HbA_1c_ level threshold by race/ethnicity							
	6.1% among all races/ethnicities	6.3% among all races/ethnicities	6.5% by race/ethnicty					6.7% among all races/ethnicities
			Asian, non-Hispanic	Black, non-Hispanic	Hispanic	White, non-Hispanic	All races/ethnicities	
EGH								
Rate (95% CI) per 1000 PY	0.64 (0.59-0.68)	0.72 (0.67-0.77)	0.60 (0.47-0.75)	0.82 (0.66-1.00)	0.63 (0.52-0.72)	0.83 (0.77-0.90)	0.77 (0.71-0.83)	0.83 (0.77-0.90)
Patients with complete follow-up, No. (%)	393 674 (79.5)	312 833 (79.1)	35 223 (81.9)	34 195 (82.6)	94 324 (77.2)	90 052 (79.7)	261 322 (78.8)	229 341 (78.6)
Risk among patients with complete follow-up, % (95% CI)	0.21 (0.20-0.23)	0.24 (0.22-0.26)	0.20 (0.16-0.25)	0.27 (0.22-0.33)	0.21 (0.18-0.24)	0.33 (0.30-0.37)	0.26 (0.24-0.28)	0.28 (0.26-0.30)
DEC								
Rate (95% CI) per 1000 PY	0.63 (0.57-0.70)	0.86 (0.76-0.96)	0.67 (0.44-0.98)	0.87 (0.58-1.25)	0.77 (0.60-0.97)	1.53 (1.27-1.82)	1.01 (0.89-1.15)	1.17 (1.01-1.35)
Patients with complete follow-up, No. (%)	166 562 (79.1)	105 906 (78.6)	11 556 (79.9)	9103 (82.3)	24 459 (75.7)	23 131 (79.1)	69 744 (77.7)	48 090 (76.6)
Risk among patients with complete follow-up, % (95% CI)	0.21 (0.19-0.23)	0.29 (0.26-0.32)	0.22 (0.15-0.33)	0.29 (0.19-0.42)	0.26 (0.20-0.33)	0.51 (0.43-0.61)	0.34 (0.30-0.39)	0.40 (0.34-0.46)
IHC								
Rate (95% CI) per 1000 PY	0.78 (0.61-0.98)	1.21 (0.97-1.48)	0.72 (0.32-1.42)	0.83 (0.35-1.71)	0.84 (0.48-1.37)	2.37 (1.75-3.14)	1.37 (1.07-1.72)	1.82 (1.39-2.33)
Patients with complete follow-up, No. (%)	29 030 (83.2)	23 074 (82.9)	3173 (84.6)	2213 (84.0)	5075 (82.1)	5838 (82.5)	16 562 (82.8)	10 231 (81.6)
Risk among patients with complete follow-up, % (95% CI)	0.25 (0.20-0.32)	0.39 (0.32-0.48)	0.23 (0.11-0.48)	0.27 (0.12-0.59)	0.28 (0.16-0.46)	0.77 (0.58-1.0)	0.50 (0.40-0.62)	0.80 (0.65-0.99)

Abbreviations: DEC, diabetes-excluded cohort; EGH, elevated glycated hemoglobin; HbA_1c_, glycated hemoglobin; IHC, confirmed index hyperglycemia cohort; PY, person-years.

### Number and Proportion of PDAC Cases

The total number PDAC cases in the base cohort was 1041. The number of PDAC cases that developed in 3 years and the percentage of PDAC cases within the base cohort captured in each cohort is shown in eTable 1 in the Supplement. The EGH cohort, DEC, and IHC contained 641 (61.6%) to 838 (80.5%), 191 (18.3%) to 351 (33.7%), and 61 (5.9%) to 91 (8.7%) of all the PDAC cases, respectively.

### Timing and Stage of Pancreatic Cancer

The median (IQR) time to cancer diagnosis ranged from 246 (77-587) days to 456 (157-793) days from the time of elevated HbA_1c_ level. The median time to cancer diagnosis and percentage of cancers diagnosed in the 1 and 2 years following the index laboratory test for each cohort are presented in Table 3. A large proportion of the cancers that were diagnosed were identified within the first year from index laboratory abnormality (eg, 141 [59.2%], 42 [56.8%], and 307 [45.5%] of all cancers in the DEC_6.5%_, IHC_6.5%_, and EGH_6.5%_ cohort, respectively) (Table 3).

**Table 3.** Median Time to Cancer Diagnosis and Percentage Who Developed Pancreatic Cancer in 1 Year and 2 Years Among All Pancreatic Ductal Adenocarcinoma Cases, by Cohort

Cohort	HbA_1c_ threshold			
	>6.1%	>6.3%	>6.5%	>6.7%
Elevated HbA1c cohort				
Time to diagnosis, median (IQR), d	456 (157-793)	419 (128-753)	418 (127-747)	404 (127-729)
Diagnosed within 1 y, No. (%)	364 (43.4)	641 (84.9)	307 (45.5)	299 (46.6)
Diagnosed within 2 y, No. (%)	582 (69.5)	554 (73.4)	502 (74.4)	483 (75.4)
Diabetes-excluded cohort				
Time to diagnosis, median (IQR), d	350 (107-755)	295 (84-648)	258 (88-590)	246 (77-587)
Diagnosed within 1 y, No. (%)	183 (52.1)	174 (57.2)	141 (59.2)	117 (61.3)
Diagnosed within 2 y, No. (%)	253 (72.1)	242 (79.6)	201 (84.5)	158 (82.7)
Confirmed index hyperglycemia cohort				
Time to diagnosis, median (IQR), d	317 (147-685)	315 (111-581)	286 (128-580)	270 (115-581)
Diagnosed within 1 y, No. (%)	42 (56.8)	52 (57.1)	42 (56.8)	37 (60.7)
Diagnosed within 2 y, No. (%)	58 (78.4)	79 (86.8)	68 (91.9)	53 (86.9)

Abbreviations: HbA_1c_, glycated hemoglobin; IQR, interquartile range.

A graphic depiction of stage at time of cancer diagnosis is presented in the eFigure in the Supplement. Among the 708 of 1041 patients (68.0%) with staging information available, 465 (65.7%) were diagnosed at an advanced stage (ie, III or IV). The proportion of late-stage cancer diagnoses remained consistent across cohorts. For example, in the EGH_6.5%_ cohort, 322 of 512 cases (62.9%) with a known stage were diagnosed at an advanced stage (stage III or IV). The corresponding numbers and the percentages were 127 of 191 (66.5%) and 38 of 56 (67.9%) in DEC_6.5%_ and IHC_6.5%_, respectively.

### Number of Patients Needing to Undergo Evaluation to Potentially Detect 1 Pancreatic Cancer

Table 4 presents the estimated number of cases that would need to be evaluated in a 3-year period to detect a single case of pancreatic cancer based on the cancer risk observed within each of the study cohorts (assuming 100% ability to identify an existing cancer). The estimate ranged from a low of 206 (95% CI, 160-264) in patients in IHC_6.7%_ to a high of 600 (95% CI, 540-666) in patients in the EGH_6.1%_ cohort. The number needed to undergo evaluation to identify a single case of PDAC in the base cohort was 818 (95% CI, 770-869).

**Table 4.** Number of Patients Needed to Be Evaluated During a 3-Year Period to Detect 1 Case of Pancreatic Cancer, by Cohort

Cohort	No. (95% CI), by HbA_1c_ threshold			
	>6.1%	>6.3%	>6.5%	>6.7%
Elevated HbA1c cohort	591 (552-632)	524 (488-562)	491 (456-530)	455 (421-492)
Diabetes-excluded cohort	600 (540-666)	443 (396-496)	377 (332-428)	328 (285-378)
Confirmed index hyperglycemia cohort	471 (376-592)	306 (249-375)	270 (216-339)	206 (160-264)

Abbreviation: HbA_1c_, glycated hemoglobin.

## Discussion

In this large retrospective comparative cohort study, the risk of pancreatic cancer in persons aged 50 to 84 years varied according to both the degree of elevation in HbA_1c_ level as well as timing with respect to onset of diabetes. Adoption of specific thresholds for elevation in HbA_1c_ level while applying the broadest set of cohort entry criteria without exclusion based on prior diabetes status provided the greatest sensitivity (range, 62%-80%) for detection of pancreatic cancer while still affording a 1.4- to 1.8-fold increase in cancer incidence from the base cohort. Using the same thresholds for HbA_1c_ level but applying a much more stringent definition for incident hyperglycemia yielded a 1.7- to 4.0-fold increase in cancer incidence at the expense of sensitivity (range, 6%-9%).

A major contributing factor to poor survival in pancreatic cancer is the late stage at diagnosis.^14^ Based on the relatively low incidence, it is unlikely that widespread, population-based screening would prove beneficial. However, a targeted approach to screening patients at increased risk offers the potential for early detection, thereby improving survival. While efforts to apply screening in patients at increased risk based on family history or genetic predisposition appear promising,^15,16^ these inherited forms account for a very small percentage (ie, 3% to 5%) of pancreatic cancer cases. Therefore, attention has turned to evaluating a broader segment of the population. In particular, there is a growing body of evidence suggesting that patients aged 50 years or older with incident diabetes constitute an additional high-risk population for pancreatic cancer.^17,18,19^ While previous attempts have focused on identifying a high-risk subgroup based on the identification of new-onset diabetes,^6,8^ the present study expands on previous research by providing estimates of pancreatic cancer incidence across a range of newly established hyperglycemia, including levels that would qualify as prediabetes. The present study also highlighted potential differences in the association of new-onset hyperglycemia and the risk of pancreatic cancer based on race/ethnicity that have not been reflected in recent attempts to develop risk-prediction models to further identify high-risk subgroups. Accounting for these differences in future approaches to risk stratification is an important step to avoid potentially exacerbating existing disparities in pancreatic cancer.^20,21,22^

The observed rates of pancreatic cancer among patients with hyperglycemia were lower than prior estimates based on patients with new-onset diabetes. Population-based estimates of pancreatic cancer incidence among individuals with new-onset diabetes have focused on the first 3 years after diagnosis. These estimates have varied from 0.4% to 1.0%.^6,8^ An explanation for the discrepancy has been that studies incorporating diabetes diagnosed based on glycemic parameters tended to report increased rates of pancreatic cancer compared with reliance on diagnosis codes, potentially because of a delay in clinical diagnosis despite abnormal glucose or HbA_1c_ values.^23^ Although we used glycemic criteria in the present study, estimates for 3-year risk of pancreatic cancer were closer to 0.4% among patients with confirmed incident diabetes using a threshold of 6.5% for HbA_1c_ level. There are several potential explanations for the lower estimate observed in the present study. First, we focused exclusively on HbA_1c_ levels as the glycemic parameter of interest, rather than on fasting glucose, which was the predominant measure used in previous studies reporting higher rates of pancreatic cancer.^8,17^ We focused on HbA_1c_ level as a measure of hyperglycemia because of the increased use of this parameter in the KPSC health system following the 2010 guideline from the American Diabetes Association, which included elevation in HbA_1c_ level as part of the diagnostic criteria for diabetes.^10^ Second, the current study population was substantially more racially/ethnically diverse compared with those in prior studies.^7,8,24^

### Limitations and Strengths

There were several important limitations to the present study. First, as previously noted, we limited the analysis to evaluation of HbA_1c_ level. It is conceivable that cancer rates would vary if other measures of hyperglycemia were included. Second, we did not examine the role of antihyperglycemic medications, which could have played a role in determining glycemic status in the study cohorts that included patients with a history of diabetes. This was beyond the scope of the study, given that exposure to antidiabetic medications was an exclusionary factor for most of the cohorts included in the analysis. Additionally, selection bias may have occurred based on clinician-related decisions to measure HbA_1c_ level, given that patients with this measure may have had poorer health at baseline or been more likely to have established diabetes (as evidenced by the relatively elevated median HbA_1c_ level in the base cohort). As a result, we pursued a variety of methods to exclude patients with evidence of prior diabetes diagnosis from the analytic study cohorts. In addition, because cases from the California death file did not have histologic diagnoses available, it is possible that a small proportion of non–ductal adenocarcinoma subtypes of pancreatic cancer may have been included in the analysis. Furthermore, while we have provided an estimate of the number of patients needed to undergo investigation to detect a case of pancreatic cancer under a variety of scenarios, it was not possible to provide a more precise estimate of the number needed to screen given lack of data on the potential effectiveness of currently available early detection strategies based on either cross-sectional imaging or endosonography to improve survival in pancreatic cancer.^25,26,27^

Despite the study limitations, the present study has multiple strengths. These include the relatively large sample size and racially/ethnically diverse study population. In addition, the setting of an integrated health care system enabled accurate assessment of prior diabetes status. Finally, the use of a prospectively maintained cancer registry as well as a state-wide death index further ensured accurate identification of patients who developed pancreatic cancer.

## Conclusions

In this study, elevated HbA_1c_ level among individuals aged 50 to 85 years was associated with increased risk of pancreatic cancer. However, the risk varied by race/ethnicity, with the highest risk noted among non-Hispanic white patients with confirmed evidence of incident hyperglycemia in the absence of prior diabetes or elevated HbA_1c_ value. An increased risk of pancreatic cancer based on more stringent definitions of new-onset hyperglycemia was not observed among other racial/ethnic groups. Ultimately, the number of patients needing to undergo testing based solely on elevation in HbA_1c_ level exceeded reasonable limits based on available testing strategies and resources. Alternative approaches to risk stratification are still needed to improve early detection of pancreatic cancer.

## Notes

Supplement. **eTable.** Number of Patients with PDAC and Percentage of PDAC Cases Among 1041 PDAC Cases in Base Cohort **eFigure.** Stage at Cancer diagnosis by HbA_1c_ Level and Cohort
