# Supplementary material for: Association of Glycated Hemoglobin Levels With Risk of Pancreatic Cancer
Source: JAMA Netw Open. 2020 Jun 12;3(6):e204945. doi: 10.1001/jamanetworkopen.2020.4945 (PMC7292999; doi:10.1001/jamanetworkopen.2020.4945)
Supplement: Supplement. — eTable. Number of Patients with PDAC and Percentage of PDAC Cases Among 1041 PDAC Cases in Base Cohort eFigure. Stage at Cancer diagnosis by HbA1c Level and Cohort [file jamanetwopen-3-e204945-s001.pdf]

# Supplementary Online Content

Wu BU, Butler RK, Lustigova E, Lawrence JM, Chen W. Association of glycated hemoglobin levels with risk of pancreatic cancer. *JAMA Netw Open*. 2020;3(6):e204945. doi:10.1001/jamanetworkopen.2020.4945

**eTable.** Number of Patients with PDAC and Percentage of PDAC Cases Among 1041 PDAC Cases in Base Cohort

**eFigure.** Stage at Cancer diagnosis by HbA1c Level and Cohort

This supplementary material has been provided by the authors to give readers additional information about their work.

© 2020 Wu BU et al. *JAMA Network Open.*

eTable. Number of Patients with PDAC and Percentage of PDAC Cases Among 1041 PDAC Cases in Base Cohort

| | HgA1c Threshold | | | |
|---|---|---|---|---|
| | 6.1% | 6.3% | 6.5% | 6.7% |
| Elevated glycosylated hemoglobin (EGH) | 838 (80%) | 755 (72%) | 675 (64%) | 641 (61%) |
| Diabetes excluded cohort (DEC) | 351 (34%) | 304 (29%) | 238 (23%) | 191 (18%) |
| Confirmed index hyperglycemia cohort (IHC) | 74 (7%) | 91 (9%) | 74 (7%) | 61 (6%) |

© 2020 Wu BU et al. *JAMA Network Open.*

eFigure. Stage at Cancer diagnosis by $HbA_{1c}$ Level and Cohort

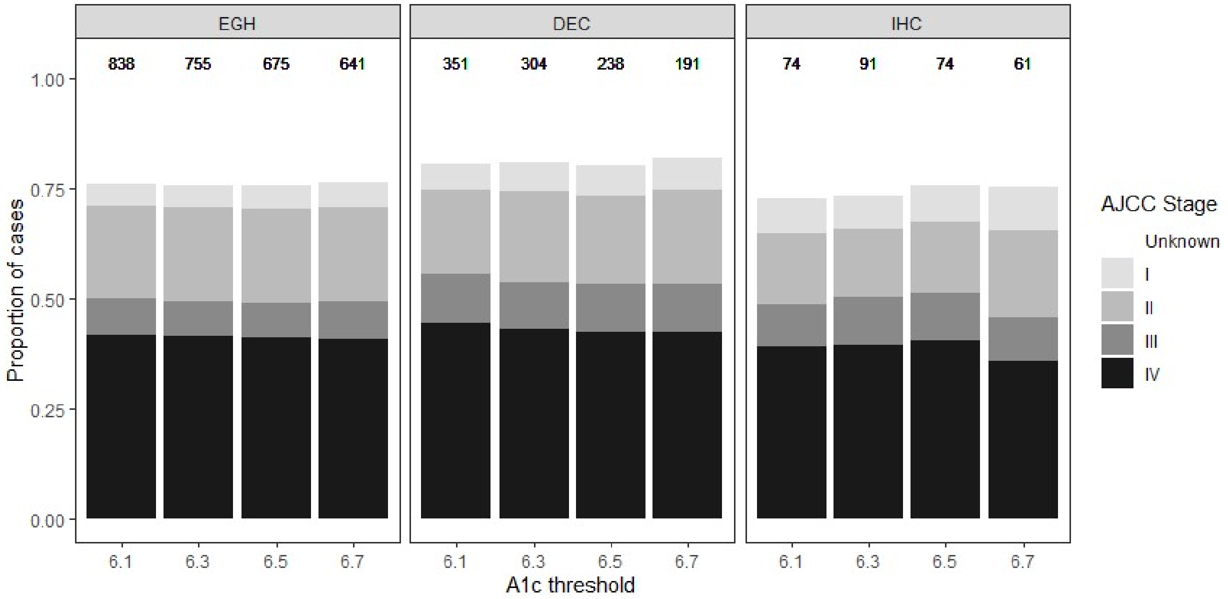


AJCC= American Joint Committee on Cancer

© 2020 Wu BU et al. *JAMA Network Open.*
